# Resurfacing hemiarthroplasty *versus* stemmed hemiarthroplasty for glenohumeral osteoarthritis: a meta-analysis

**DOI:** 10.1186/s42836-020-00045-5

**Published:** 2020-09-01

**Authors:** Baoliang Zhang, Guanghui Chen, Tianqi Fan, Zhongqiang Chen

**Affiliations:** Department of Orthopaedics, Peking University Third Hospital, No. 49 North Garden, Road, Haidian District, Beijing, 100191 China

**Keywords:** Resurfacing hemiarthroplasty, Stemmed hemiarthroplasty, Glenohumeral osteoarthritis, Meta-analysis

## Abstract

**Background:**

Though total shoulder arthroplasty (TSA) has been an acknowledged treatment option for glenohumeral osteoarthritis, resurfacing hemiarthroplasty (RHA) and stemmed hemiarthroplasty (SHA) may be preferred in some circumstances by surgeons, especially for treating young or active patients. However, decision-making between the RHA and SHA is controversial. Therefore, we conducted a meta-analysis to systematically compare two surgical procedures in terms of postoperative functional outcomes, range of motion (ROM), pain relief, complication rates, risk of revision.

**Methods:**

The PubMed, Embase, Web of Science and Cochrane Library were searched from inception to January 1, 2020, for all articles that compared the clinical effectiveness and safety of RHA with SHA. All eligible studies were selected based on certain screening criteria. Two investigators independently conducted the quality assessment and extracted the data. Fixed-effect and random-effect models were used for pooled results according to the degree of heterogeneity. All statistical analyses were performed by employing Stata software 14.0.

**Results:**

A total of six comparative studies involving 2568 shoulders (1356 RHA and 1212 SHA) were included in the final analysis. Patients were followed up for at least 1 year in each study. Pooled results showed that RHA was associated with a better visual analog scale (SMD 0.61, *p* = 0.001) but higher revision rates (OR 1.50, *p* = 0.016) when compared to SHA. There were no significant differences in functional outcomes, such as Constant-Murley score (SMD 0.06, *P* = 0.878), American Shoulder and Elbow Surgeons score (SMD 0.05, *P* = 0.880), Western Ontario Osteoarthritis of the Shoulder index (SMD 0.43, *p* = 0.258) and quick-Disabilities of the Arm, Shoulder and Hand score (SMD 0.06, *p* = 0.669). In addition, no differences were observed in forward flexion (SMD 0.16, *p* = 0.622), external rotation (SMD -0.17, *P* = 0.741) and overall complication rates (OR 1.42, *p* = 0.198).

**Conclusion:**

This is the first meta-analysis to investigate the clinical efficacy and safety of RHA in comparison with SHA for the treatment of glenohumeral osteoarthritis. The results demonstrated that the two surgical techniques were equivalent in terms of postoperative functional outcomes and complication rate. However, RHA provided greater pain relief but posed a higher risk for revision than SHA. More high-quality studies with long-term follow up are warranted to give more convincing evidence.

## Introduction

Glenohumeral osteoarthritis (GHOA), primary or secondary, is characterized by progressive wear of articular cartilage and eventual loss of joint movement functions [1–3]. It affects one third of the world’s population, especially those over 60 years [1]. With population aging around the globe, its prevalence has been on the rise [4]. Aggravating pain and physical restriction of the shoulder joint are two major complaints [5]. Generally, initial intervention is conservative, including activity modification, analgesic medication and physical therapy [3]. When symptoms become severe and refractory, joint replacement may be a feasible choice for most patients [6]. To date, a variety of surgical techniques have been adopted clinically, including total shoulder arthroplasty (TSA), reverse shoulder arthroplasty (RSA), stemmed hemiarthroplasty (SHA) or resurfacing hemiarthroplasty (RHA).

**Fig. 1:**
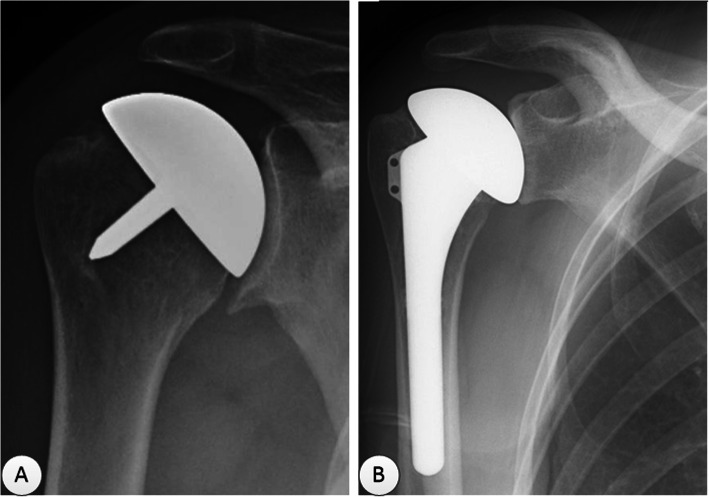
Anteroposterior radiographs (postoperation) for the treatment of GHOA. **a** resurfacing hemiarthroplasty (RHA); **b** stemmed hemiarthroplasty (SHA)

TSA has been considered the standard surgical procedure for patients with primary GHOA because of its outstanding clinical results with respect to pain relief and restoration of range of motion (ROM), especially postoperative improvement of quality of life [2, 7–9]. Nevertheless, several concerns about TSA linger, such as limited prosthesis longevity and frustrating glenoid loosening, along with technically demanding revisions, which may cause pain and loss of function [3, 10–13]. To address these problems, hemiarthroplasty (HA) is introduced as a viable option for the treatment of shoulder disorders [14–18], and is widely applied in various age groups [19–21]. It only replaces the humeral side and avoids potential complications associated with glenoid implantation [1, 22], as shown by patient-reported outcomes [23–25]. Therefore, HA may be preferred for high-demanding athletes and laborers [26]. Clinically, conventional HA, *i.e.*, SHA (Fig. 1a), is commonly performed but has some disadvantages, such as erosion of the native glenoid, loss of joint space and posterior humeral subluxation, which contribute to poor satisfaction and high revision rates in many cases, especially in young population [27–29]. To reduce the aforementioned potential risks, the Copeland Mark 3 resurfacing arthroplasty was, for the first time, introduced in 1993 as the first-generation RHA (Fig. 1b), which is a less invasive humeral head surface replacement with minimal bone resection. Subsequently, some studies reported that RHA could provide recovery of pain-free functional motion and facilitate the revision to TSA or RSA despite high complication rate during long-term follow-ups [30–37].

However, direct high-quality studies comparing RHA and SHA in light of clinical efficacy and safety are still scanty, and, as a result, no consensus has been reached on the selection between the two procedures. Several national registry studies compared patient-reported outcomes between RHA and SHA but no significant differences were observed [38–40]. Moreover, a published review comprehensively discussed their indications, clinical assessment, patients’ satisfaction and postoperative complications but was restricted to descriptive analysis [41]. To the best of our knowledge, there was no meta-analysis that provided more reliable evidence on this issue. Therefore, we made the best of currently available evidence and performed a meta-analysis to systematically and statistically determine postoperative functional outcomes, complication rates, revision risks of RHA for the treatment of GHOA as compared to SHA.

## Methods

### Search strategy

The meta-analysis was performed in accordance with the Preferred Reporting Items for Systematic Reviews and Meta-Analyses (PRISMA) guidelines [42]. Two independent reviewers systematically searched the electronic databases, including PubMed, Cochrane library, EMBASE, Web of Science until January 1, 2020 for relevant studies comparing the outcomes of RHA and SHA. The following search terms, including “resurfacing” OR “resurfacing hemiarthroplasty” OR “resurfacing shoulder hemiarthroplasty” OR “humeral head resurfacaing” OR “RHA” OR “RH” OR “HHR” AND “hemiarthroplasty” OR “stemmed hemiarthroplasty” OR “stemmed shoulder hemiarthroplasty” OR “SHA” OR “SH”, were used in all searches. Medical Subject Headings (MeSH) and Emtree terms were used in various combinations to retrieve all the potentially-relevant studies. All titles, abstracts, and full texts were screened independently by 2 reviewers (Z.B.L and C.G.H.). Disagreements were resolved by arriving at a consensus through comparing notes.

### Inclusion and exclusion criteria

The inclusion criteria for this review were as follows: (1) study population: adult patients with primary or secondary shoulder osteoarthritis; (2) interventions: RHA (investigative group) *verse* SHA (control group); (3) outcome indicators (at least one of the following outcome indicators): Constant score, ASES score, WOOS score, quick-DASH, pain score, strength, maximum active range of motions (flexion, abduction, intrarotation, extrarotation), revision rate and complications; (4) study design: randomized controlled trials (RCTs) or comparative observational studies.

The exclusion criteria were (1) Single-armed follow-up studies; (2) reviews, case reports, letters and comments; (3) studies that used cadaveric specimens or animal models; (4) studies presenting incomplete or inappropriate data; (5) non-English-language studies.

### Risk of bias and quality assessment

Two reviewers independently evaluated the quality of each included study using the Cochrane Risk of Bias Tool for RCTs and the Newcastle-Ottawa Quality Assessment Scale (NOQAS) [43] for the non-randomized comparative studies. Any dispute was resolved by reaching consensus. The Cochrane Risk of Bias Tool assesses potential selection bias, reporting bias, performance bias, detection bias, attrition bias, and other sources of bias. A score of either high, low, or unclear bias is given for each domain. The NOQAS had three main items and contained 9 points. Four points were assigned to the selection of the study population, 2 points to the comparability between groups, and 3 points to the measurement of exposure factors. When the total score of a study exceeded or was equal to 6 points, we considered it to be of high-quality.

### Data abstraction

Data from all included studies were extracted and put into a standard form independently by two investigators, with disagreement resolved by discussion. The essential information was extracted as follows: (1) study characteristics: author, publication year, country, study design; (2) patients’ demographic and clinical information: population source, age, gender, surgical procedures, number of participants, number of shoulders and mean follow-up time; (3) outcome indications: Constant-Murley score (CMS), American Shoulder and Elbow Surgeons (ASES) score, Western Ontario Osteoarthritis of the Shoulder (WOOS) index, quick-Disabilities of the Arm, Shoulder and Hand (DASH) score, pain score, range of motion (ROM), number of complications and revision rate.

### Statistical analysis

All statistical tests were performed with the Stata software package (Ver. 14.0). The odds ratio (OR) and associated 95% confidence intervals (CIs) were used to perform estimation for discontinuous variables, such as rate of complication. The mean difference (MD) or standard mean difference (SMD) was applied for continuous variables, including ASES score and WOOS score. For those continuous data presented as the means and range values, standard deviations (SD) were calculated using statistical algorithms [44]. The heterogeneity of the studies was assessed by the I^2^ statistic. The values 25%, 50%, and 75% corresponded to low, moderate, and high heterogeneity, respectively. A fixed-effect model was applied if I^2^ < 50%, and a random-effect model was used if I^2^ > 50%. Sensitivity analysis was performed to evaluate the stability of the results (when necessary), and subgroup analysis was conducted to obtain more specific conclusions if possible. Forest plots were used to present the results of the individual studies and respective pooled estimates of effect size. Publication bias was statistically assessed using visual inspection of the funnel plot. Statistical significance was defined as *p* values < 0.05.

## Results

### Included studies

**Fig. 2:**
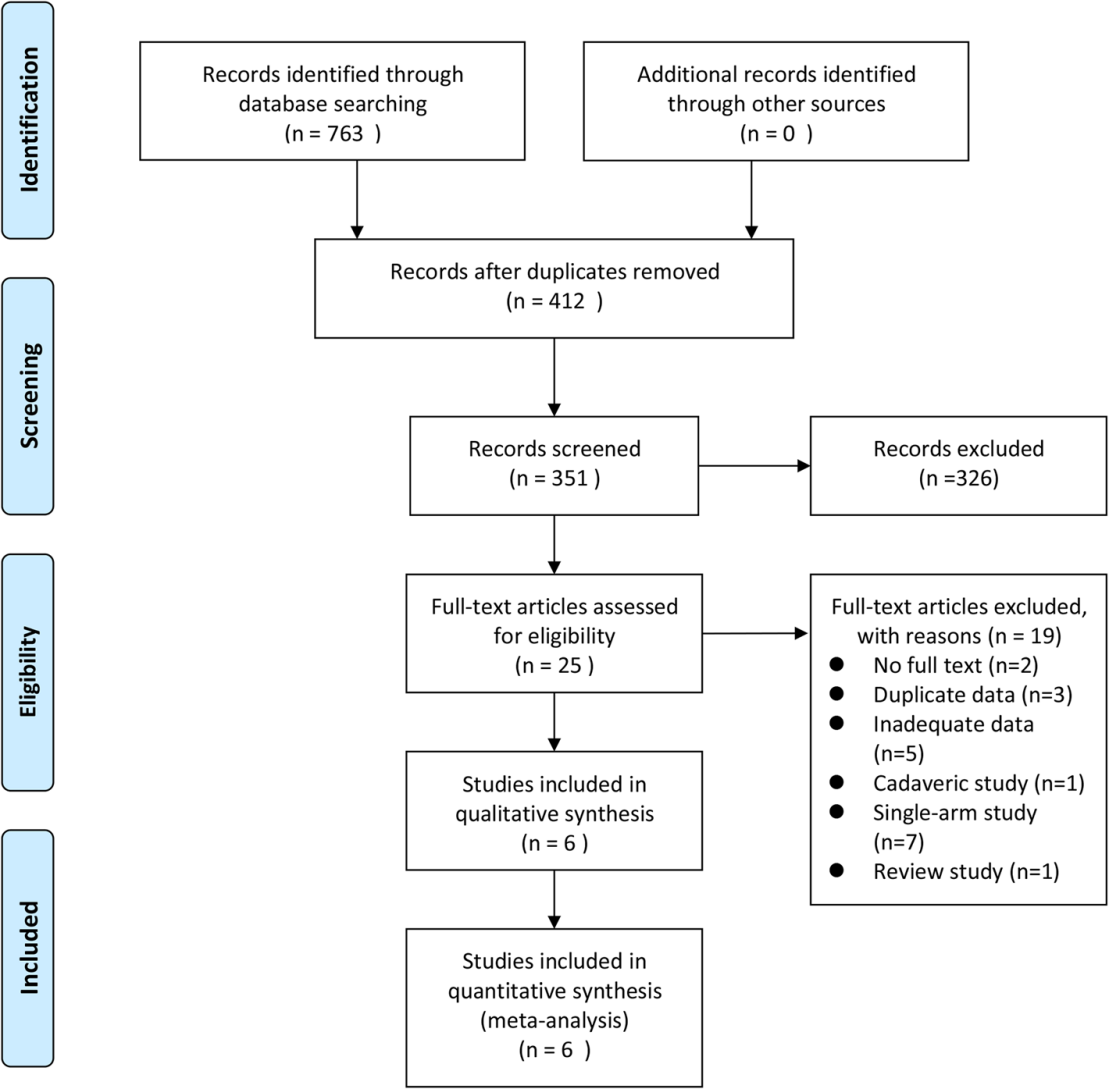
Flow diagram of the study identification and selection process

A total of 763 potentially-relevant articles were retrieved from the four electronic databases. Upon duplicate removal through Endnote software, 351 unique abstracts remained. After reading the title and abstract, 326 irrelevant studies were ruled out. After full-text review of remaining articles, 19 studies were excluded against the pre-established selection criteria. Finally, 6 eligible articles [39, 40, 45–48] that compared outcomes between RHA and SHA were included in this meta-analysis. The search strategy through the PRISMA flow diagram is detailed in Fig. 2.

### Study characteristics

The included studies involved a total of 2568 shoulders, with 1356 undergoing RHA and 1212 receiving SHA, respectively. Of the 6 studies, one was a randomized controlled trial (RCT) [47] and 5 were retrospective cohort studies [39, 40, 45, 46, 48]. Two studies [39, 47] were performed in Denmark, two [45, 48] in America, one [40] in Sweden and one [46] in France. The publication time of these studies ranged from 2013 to 2019. The mean age of the subjects ranged from 37.7 to 65.6 years in the RHA group, compared with 33.9 to 71.0 years in the SHA group. The proportion of males ranged from 19.6% to 62.3% in the RHA group, and from 22.5% to 55.3% in the SHA group. The maximum follow-up duration lasted 112.8 months and the minimum was 12 months. The study features, patients’ demographic and clinical data are listed in Table 1.

**Table 1:** The main characteristics of the included studies

Study	Country	Study design	Population	Group	No. of patients	Age (years)	Gender (M/F)	No. of shoulders	Follow-up(months)	No. of complications	No. of revisions
Hammond *et al,* 2013 [45]	USA	RCS	various^a^	R	23	37.7 ± 8.9	12/8	20	43.2 ± 14.4	12	6
				S	21	33.9 ± 9.4	9/9	20	45.6 ± 22.8	6	3
Rasmussen *et al,* 2014 [39]	Denmark	RCS	various	R	837	65 ± 11	370/467	837	≧12	NA	63
				S	259	71 ± 11	89/170	259	≧12	NA	16
Lebon *et al*, 2014 [46]	France	RCS	primary^b^	R	41	61(47–80)	20/21	41	40	10	4
				S	37	63(56–79)	18/19	37	48	6	0
Rasmussen *et al*, 2015 [47]	Denmark	RCT	primary	R	35	65.6 (40–88)	7/13	20	12	0	0
				S		69.1 (46–87)	6/14	20	12	0	0
Ödquist *et al*, 2018 [40]	Sweden	RCS	various	R	NA	67.4 ± 10.8	163/155	318	≧60	NA	37
				S	NA		380/442	822	≧60	NA	55
Fourman *et al*, 2019 [48]	USA	RCS	various	R	106	63.8 ± 9.5	66/40	120	62.4 ± 21.6	35	0
				S	47	62.5 ± 9.9	26/21	54	112.8 ± 40.8	12	2

USA the United States of America, RCS retrospective cohort studies, RCT randomized controlled trial, R resurfacing hemiarthroplasty, S stemmed hemiarthroplasty, No. numbers, M/F male/female, NA not available.

^a^Various includes inflammatory arthritis, post-traumatic arthritis, osteonecrosis, rheumatoid arthritis, and others

^b^Primary represents primary glenohumeral osteoarthritis.

### Quality assessment and risk of bias

According to the Cochrane Risk of Bias Tool, the RCT by Rasmussen *et al* [47] maintained a low risk of biases with respect to selection, performance and attrition, so it was rated as of relatively high quality (Table 2). Against the NOQAS for non-randomized studies, three studies [45, 46, 48] scored 8 points and two studies scored [39, 40] 7 points (Table 3). Therefore, methodologically, all included studies were graded as of high-quality.

**Table 2:** Methodological assessment according to the Cochrane Risk of Bias Tool for RCTs

Study	Random Sequence Generation	Allocation Concealment	Blinding of Participants and Personnel	Blinding of Outcome Assessment	Incomplete Outcome Data	Selective Reporting	OtherBias	Overall Bias
Rasmussen *et al,* 2015 [47]	Low	Low	Low	Unclear	Low	Unclear	Unclear	Low

**Table 3:** Methodological assessment based on Newcastle-Ottawa Scale for non-randomized studies

study	Selection (score)				Comparability (score)	Outcome (score)			Total score
	Represent-ativeness of the exposed cohort	Selection of the nonexpo-sed cohort	Ascertai-nment of exposure	Outcome of interest was not present at start of study	Based on the design or analysis	Assess-ment of outcome	Follow-up long enough for outcomes to occur	Adequ-acy of follow-up of cohorts	
Hammond *et al*, 2013 [45]	1	1	1	1	2	1	1	0	8
Rasmussen *et al,* 2014 [39]	1	1	1	1	1	0	1	1	7
Lebon *et al,* 2014 [46]	1	1	1	1	2	1	1	0	8
Ödquist *et al,* 2018 [40]	1	1	1	1	1	0	1	1	7
Fourman *et al,* 2019 [48]	1	1	1	1	2	1	1	0	8

### Meta-analysis results

#### Clinical outcomes

**Fig. 3:**
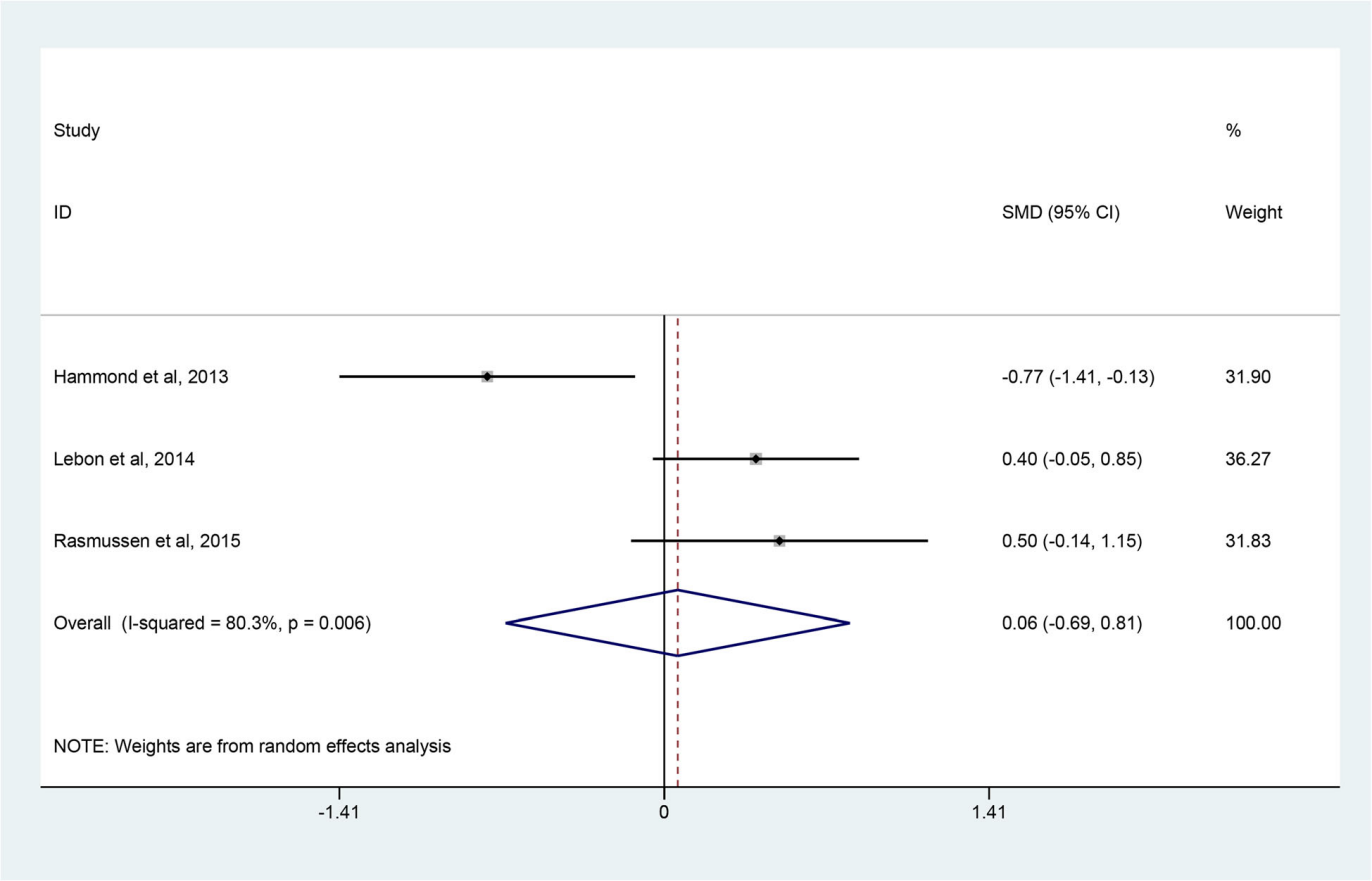
Forest plot comparing CMS scores after resurfacing hemiarthroplasty (RHA) *versus* stemmed hemiarthroplasty (SHA)

**Fig. 4:**
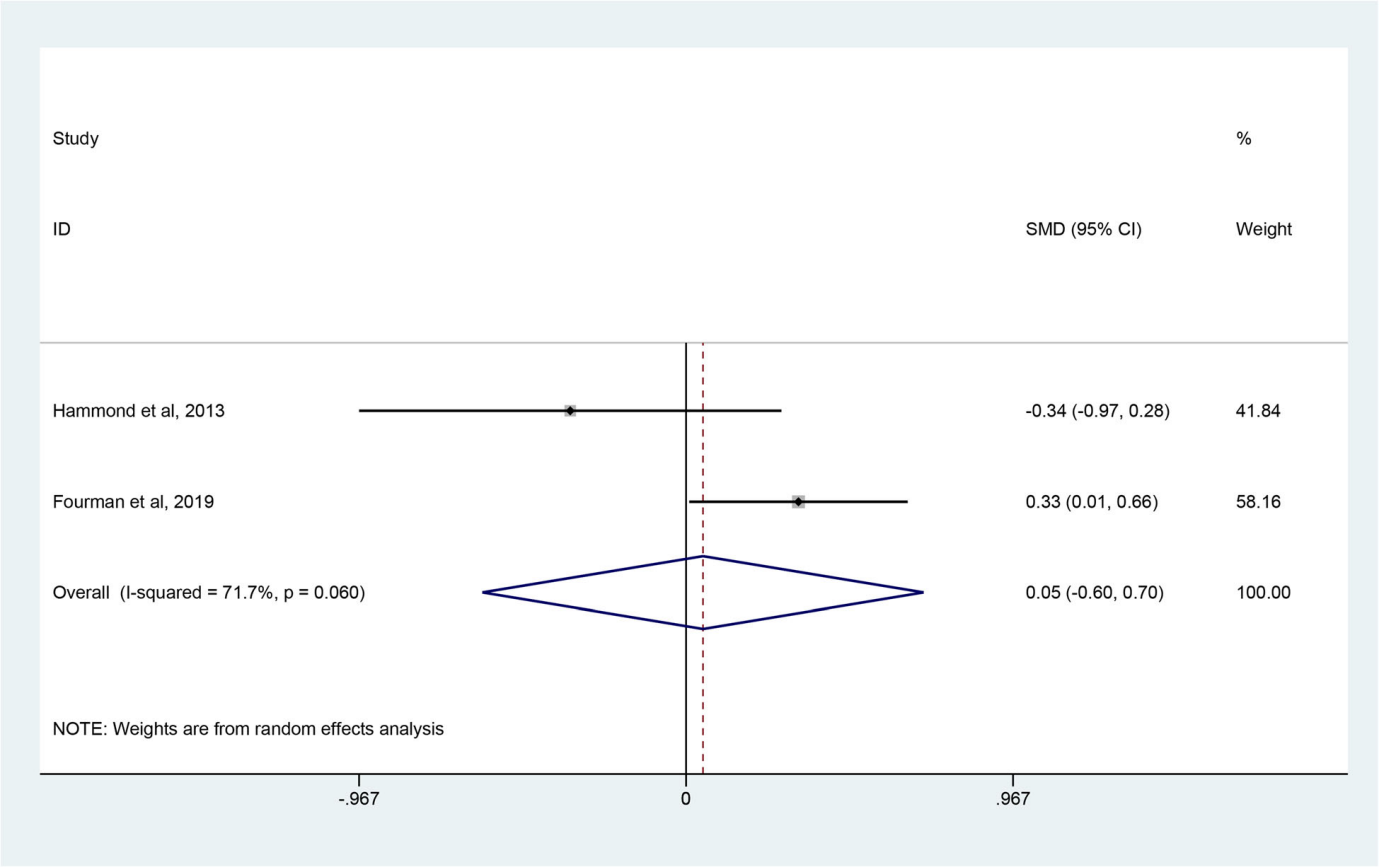
Forest plot comparing ASES scores after resurfacing hemiarthroplasty (RHA) *versus* stemmed hemiarthroplasty (SHA)

**Fig. 5:**
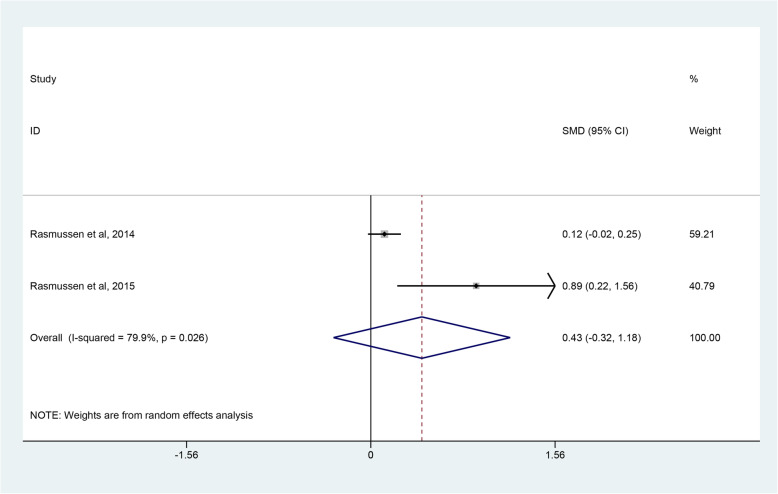
Forest plot comparing WOOS scores after resurfacing hemiarthroplasty (RHA) *versus* stemmed hemiarthroplasty (SHA).

**Fig. 6:**
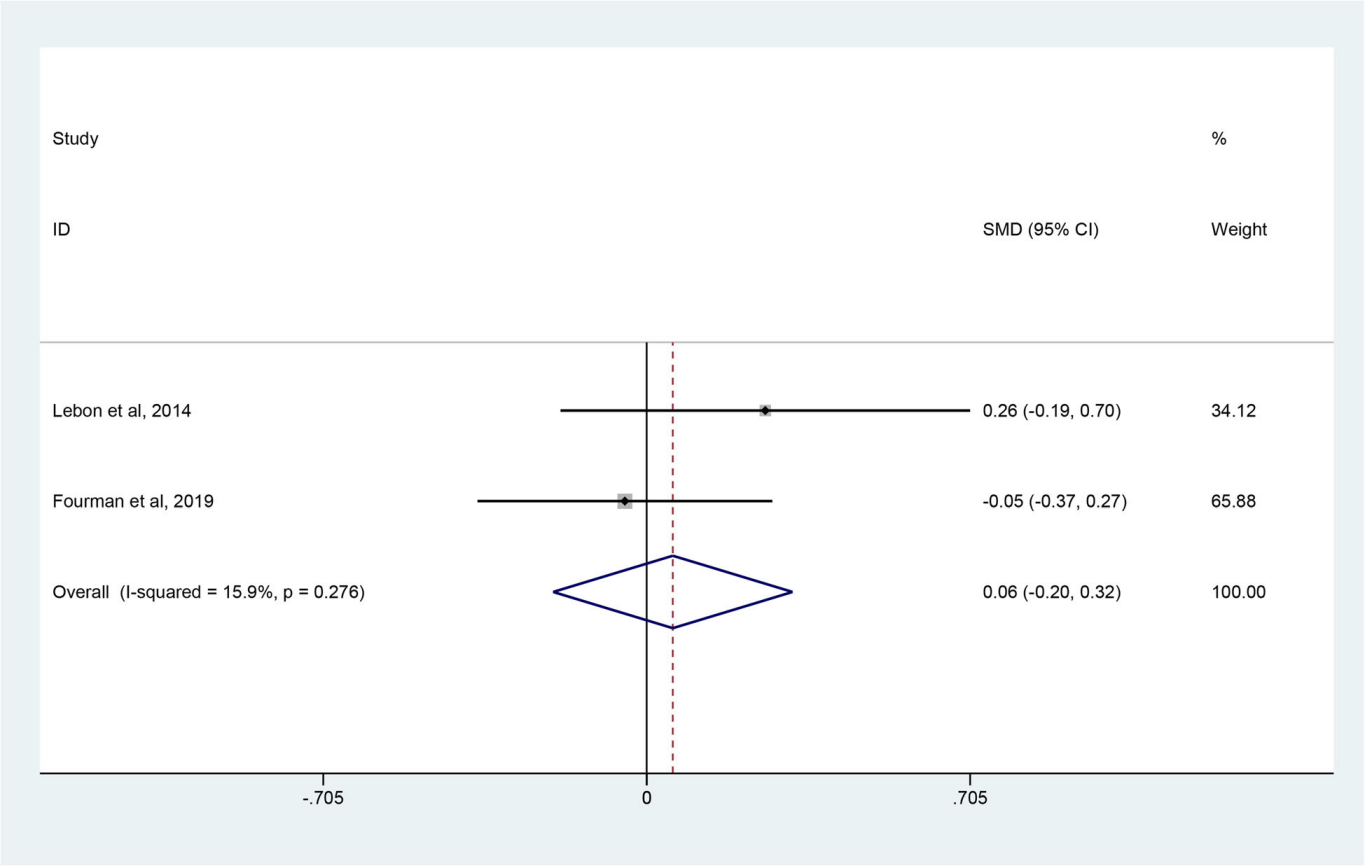
Forest plot comparing quick-DASH scores after resurfacing hemiarthroplasty (RHA) *versus* stemmed hemiarthroplasty (SHA)

The evaluation indices of clinical outcomes consisted of the CMS, ASES, WOOS and quick-DASH scores, which represent overall patient-reported functional results or satisfaction after operations. Three studies [45–47] covering 81 shoulders undergoing RHA and 77 shoulders undergoing SHA reported the CMS. Pooled results showed no significant difference in CMS between the RHA and SHA group (SMD, 0.06; 95% CI − 0.69 to 0.81; *p* = 0.878, I^2^ = 80.3%) (Fig. 3). Two studies [45, 48] involving 140 shoulders receiving RHA and 74 shoulders undergoing SHA used ASES scores to assess the outcomes between the 2 groups with no clearly significant difference observed (SMD 0.05; 95% CI − 0.06 to 0.07; *p* = 0.880, I^2^ = 71.7%) (Fig. 4). Two studies [39, 47] provided WOOS scores. They included 857 shoulders treated with RHA and 279 shoulders with SHA. There was no significant difference in WOOS scores between the 2 groups (SMD 0.43; 95% CI, − 0.32 to 1.18; *p* = 0.258, I^2^ = 79.9%) (Fig. 5). The random-effect model was used in the above-mentioned three pooled analyses with high heterogeneity. In addition, when two studies [46, 48] involving quick-DASH scores were combined, no significant difference was observed either between the groups with low heterogeneity (SMD 0.06; 95% CI, − 0.20 to 0.32; *p* = 0.669, I^2^ = 15.9%) (Fig. 6).

#### Range of motion and pain score

**Fig. 7:**
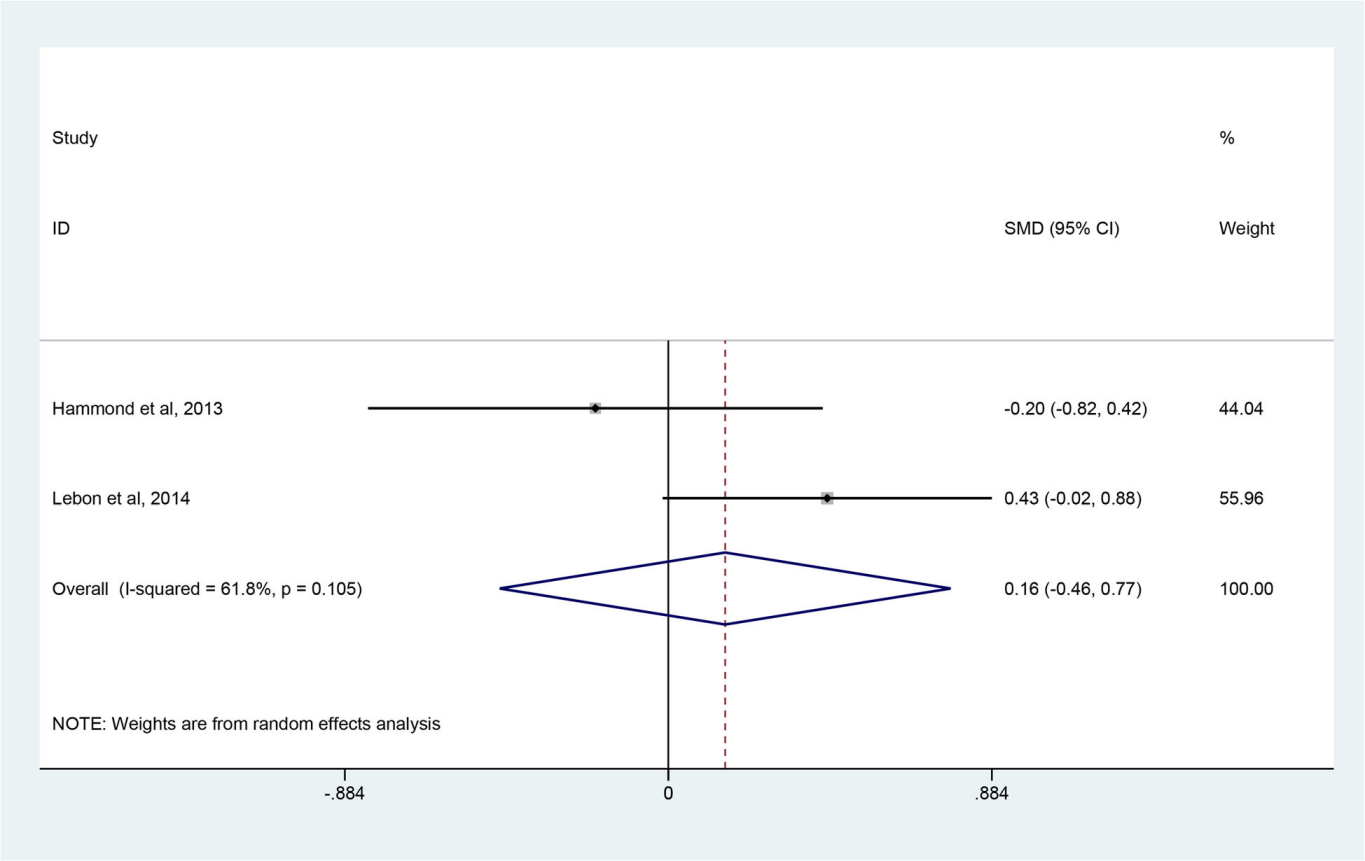
Forest plot comparing the forward flexion after resurfacing hemiarthroplasty (RHA) stemmed hemiarthroplasty (SHA)

**Fig. 8:**
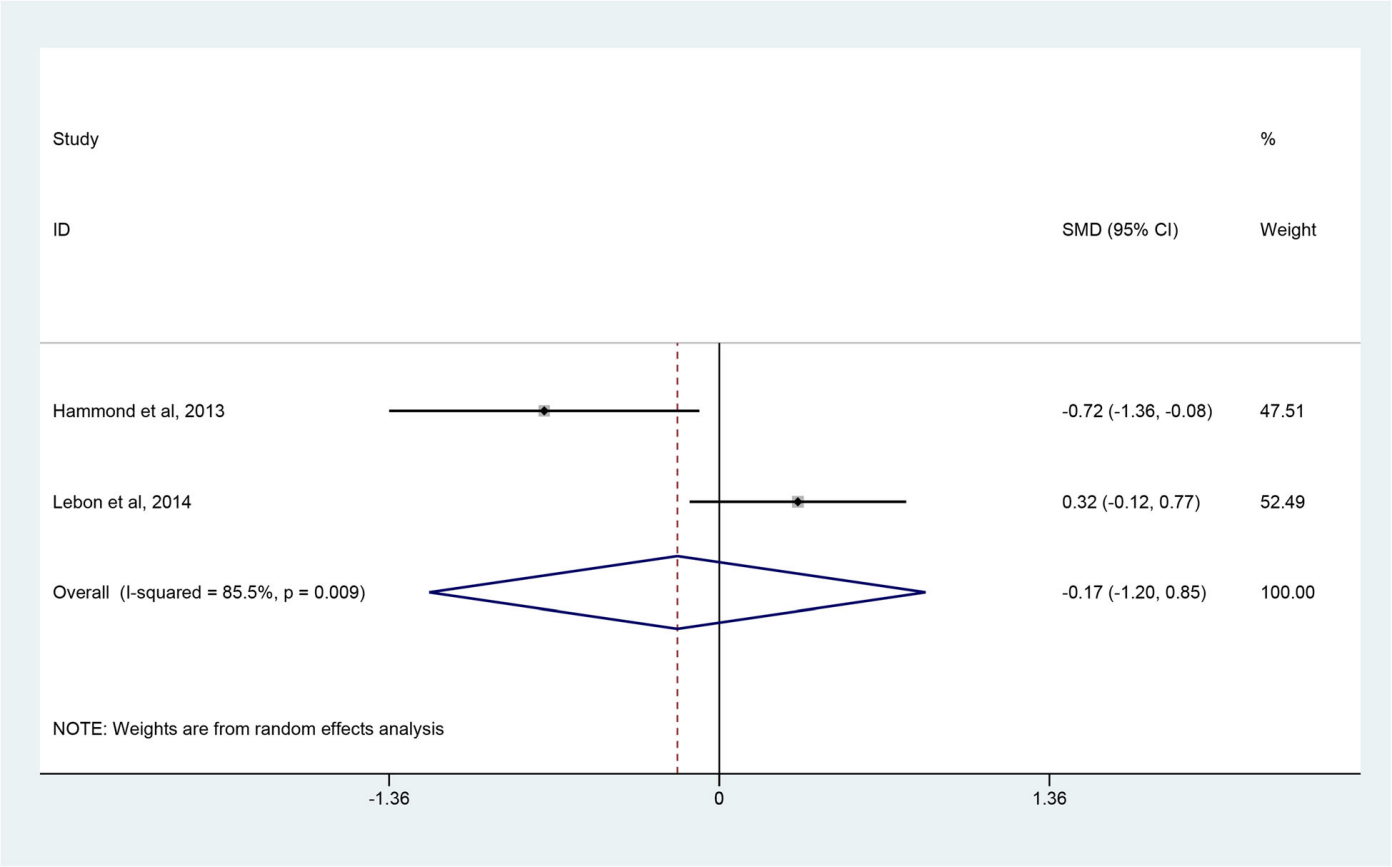
Forest plot comparing the external rotation after resurfacing hemiarthroplasty (RHA) *versus* stemmed hemiarthroplasty (SHA)

**Fig. 9:**
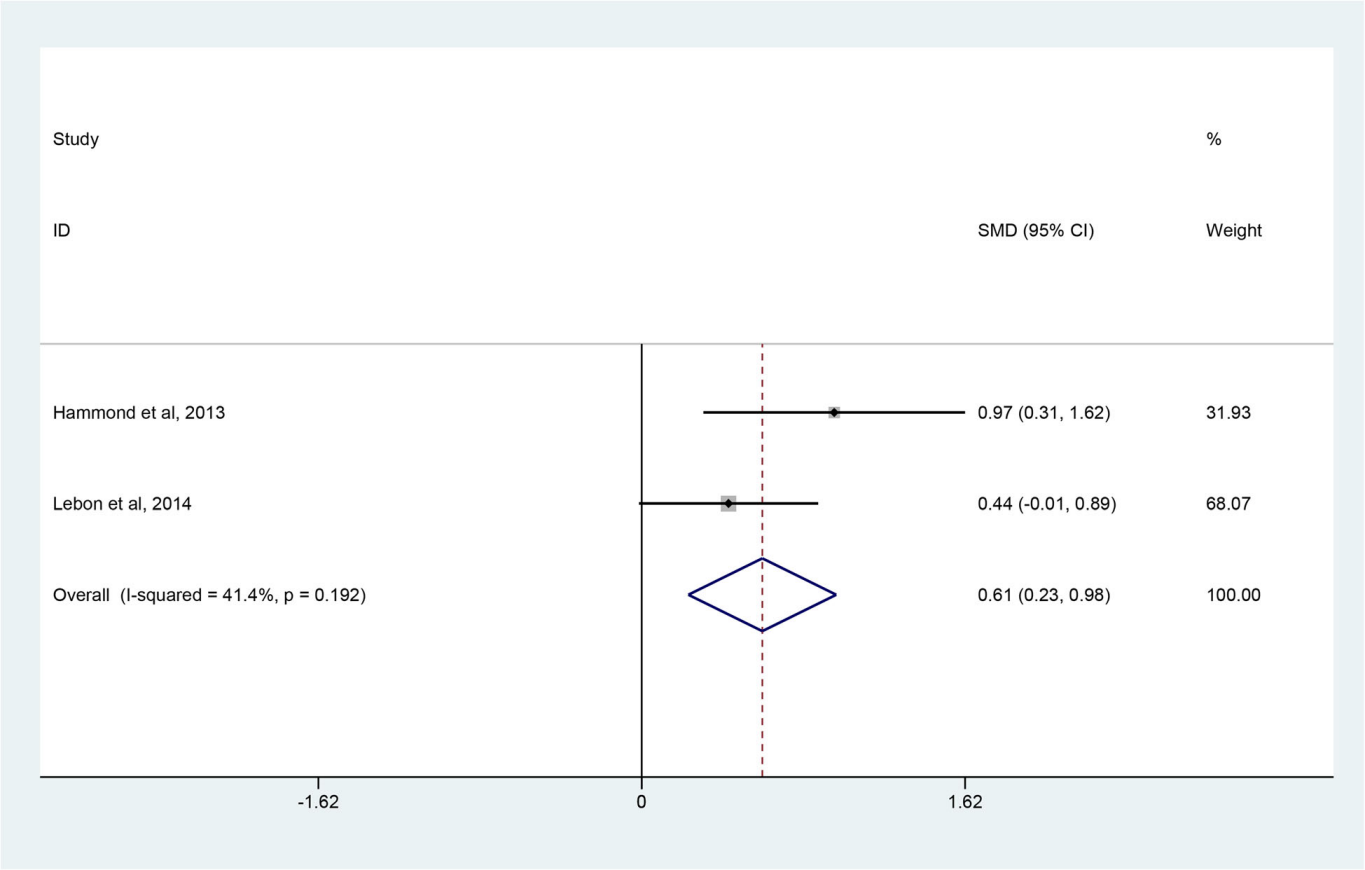
Forest plot comparing VAS scores after resurfacing hemiarthroplasty (RHA) versus stemmed hemiarthroplasty (SHA)

Four studies [45–48] reported postoperative range of motion, but only two studies [45, 46] with homologous data could be combined to assess the degree of forward flexion and external rotation. The degree of internal rotation could not be calculated due to insufficient data. Through combining the two studies with 61 RHA and 57 SHA procedures, quantitative analysis revealed that there were no significant differences in the degree of forward flexion (SMD 0.16; 95% CI − 0426 to 0.77; *p* = 0.622, I^2^ = 61.8%) (Fig. 7) and external rotation (SMD -0.17; 95% CI − 1.20 to 0.85; *p* = 0.741, I^2^ = 85.5%) (Fig. 8). Due to a high heterogeneity, the random-effect model was used. Four studies [45–48] employed three different scoring scales to evaluate postoperative pain of patients while only two studies [45, 46] that reported VAS scores were included. The pooled results demonstrated that patients in the SHA group achieved better results than those in the RHA group (SMD 0.61; 95% CI 0.23 to 0.98; *p* = 0.001, I^2^ = 41.4%) (Fig. 9).

#### Complication rates

**Fig. 10:**
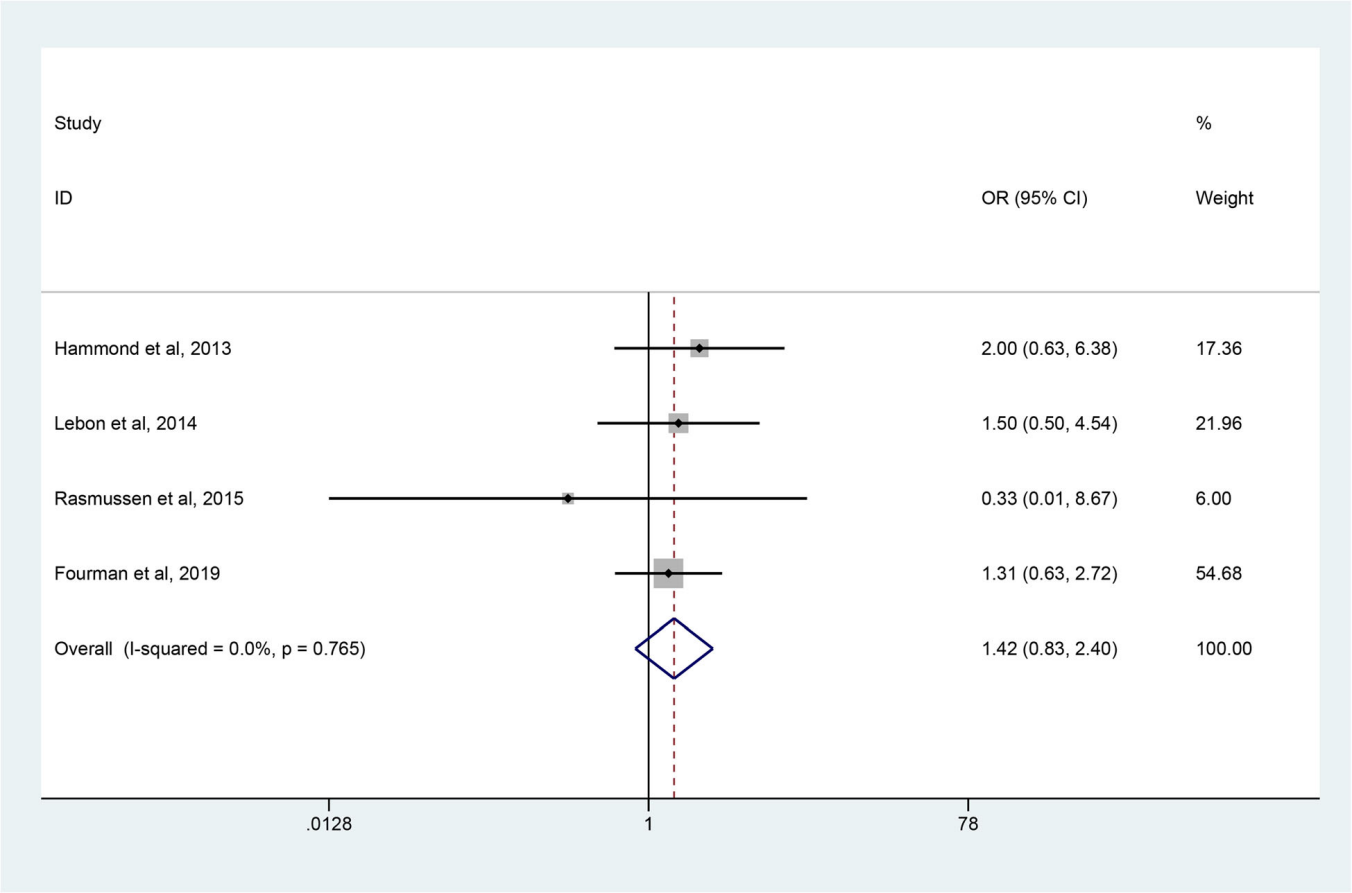
Forest plot comparing complication rates after resurfacing hemiarthroplasty (RHA) *versus* stemmed hemiarthroplasty (SHA)

The data about overall postoperative complications were extracted from four studies [45–48], which included 201 shoulders in the RHA group and 131 shoulders in the SHA group. The meta analysis found that there was no significant difference in complication rates between the two groups (OR 1.42; 95% CI 0.83 to 2.40, *p* = 0.198) without heterogeneity (*p*  = 0.756, I^2^ = 0%) (Fig. 10).

#### Revision rate

**Fig. 11:**
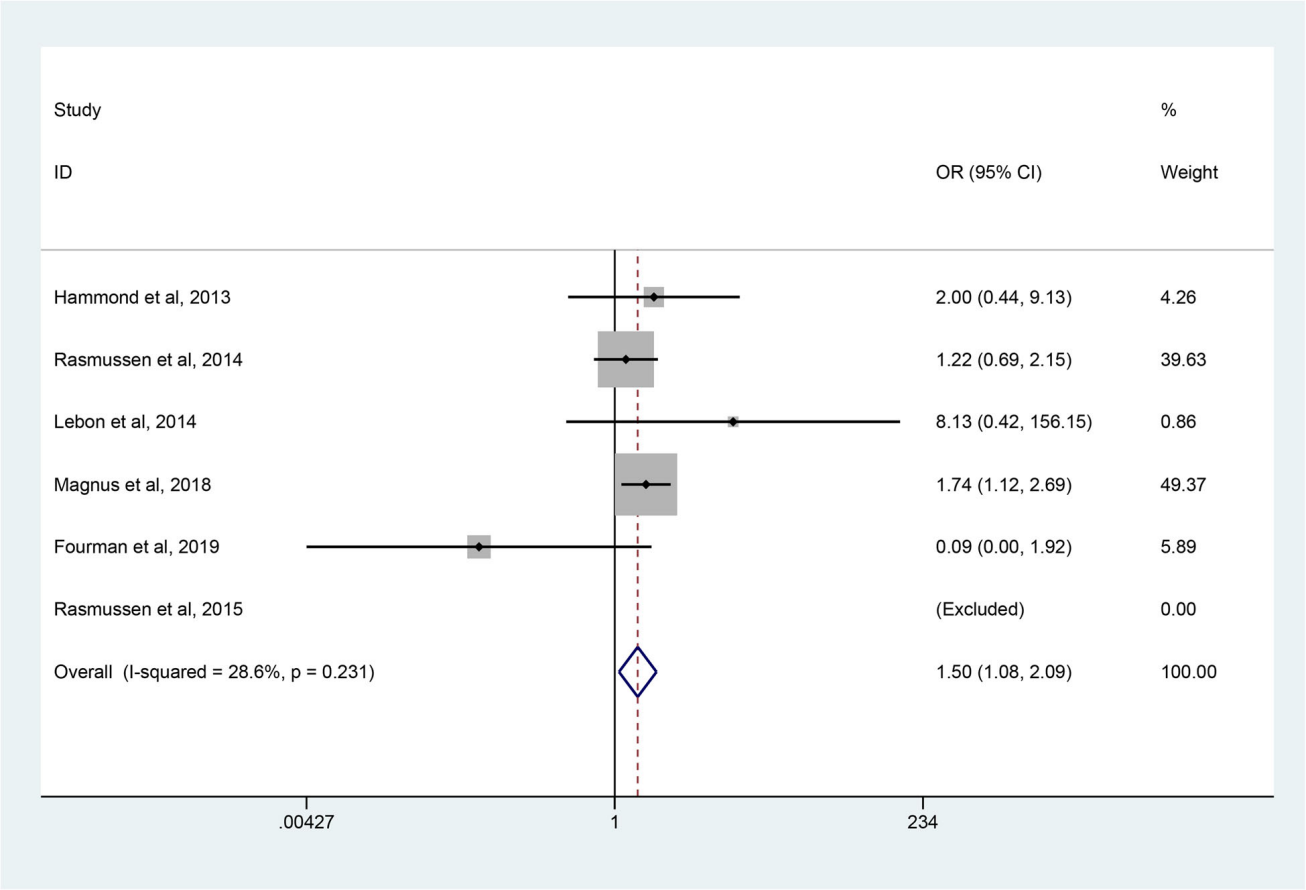
Forest plot comparing revision rates after resurfacing hemiarthroplasty (RHA) *versus* stemmed hemiarthroplasty (SHA)

The revision rates were reported or calculated in all studies [39, 40, 45–48], involving a total of 1356 RHA and 1212 SHA procedures. The pooled data showed that the revision rate after RHA was higher than that after SHA (OR 1.50; 95% CI 1.08–2.09, *p* = 0.016), with a low heterogeneity (*p* = 0.231, I^2^ = 28.6%) (Fig. 11). Therefore, a fixed effect model was applied.

### Descriptive analysis

The study by Rasmussen *et al* [47] and the other by Fourman *et al* [48] also compared postoperative pain between two groups using CMS pain subscore and ASES pain subscore, respectively. Interestingly, both results exhibited that the patients undergoing RHA out-performed those undergoing SHA in terms of pain improvement (*P* < 0.05), which were consistent with the meta-analysis results with respect to VAS.

Only one RCT by Rasmussen *et al* [47], involving 20 shoulders undergoing RHS and 20 undergoing SHA, compared the operative time. The mean operating time was 80 min (range 56–103) for SHA and 52 min (range 34–80) for RHA, with the difference being statistically significant (95% CI 18.7–36.7, *p* < 0.001).

What is more, some functional indicators, such as internal rotation, strength, activities of daily living (ADL), Subject Shoulder Value (SSV), Single Assessment Numeric Evaluation (SANE) score, Neer satisfaction score and Simple Shoulder Test (SST) were not combined due to the lack of homologous data or data being reported by a single study. However, there existed no significant differences regrading these indicators between the RHA and SHA group in each original study.

### Sensitivity analysis and publication bias

**Fig. 12:**
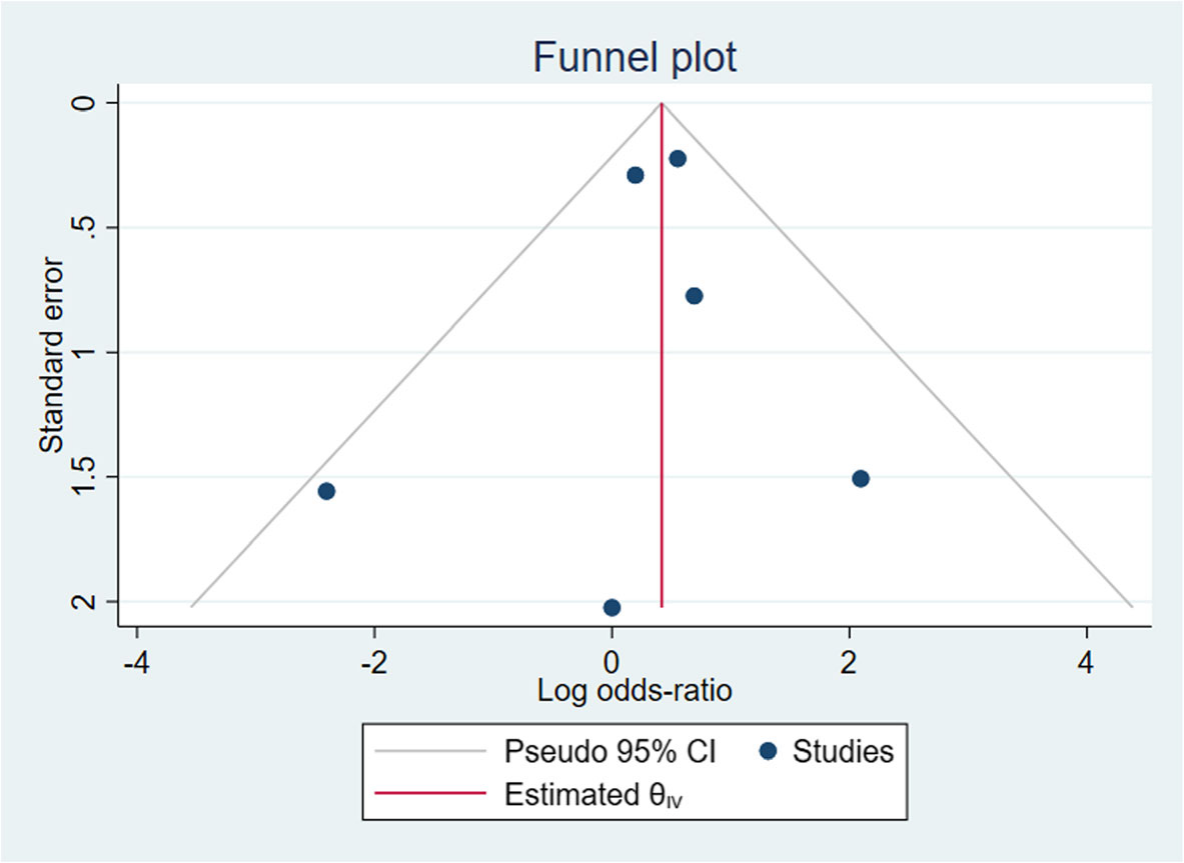
Funnel plots showing no publication bias for the revision rate

**Fig. 13:**
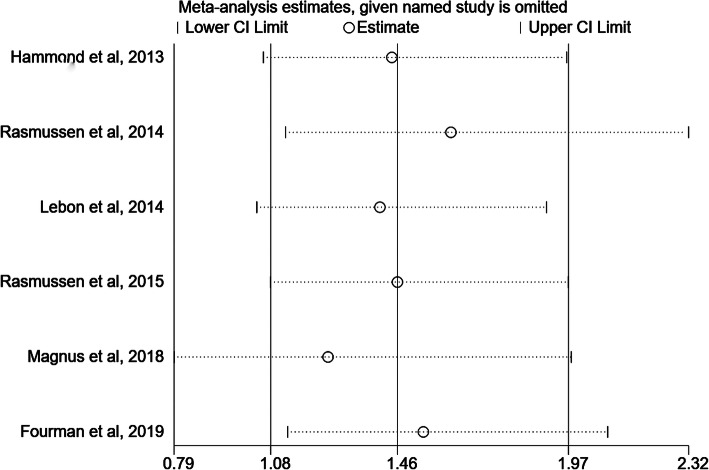
Sensitivity analysis for revision rate in all included studies

The outcome indicators, which were included in more than five studies, were required to allow for a sensitivity analysis. These outcome indicators included the complication rate and revision rate. We found that the pooled results for both indicators could be considered, in general, robust by using the one-by-one elimination method. In particular, when removing the study by Fourman *et al*, the heterogeneity decreased to 0% but the pooled results for revision rate did not change substantially (OR 1.59; 95% CI 1.13–2.23; *p* = 0.007, Fig. 12). Besides, a publication bias analysis was performed. The visual inspection of the funnel plots seemed basically symmetrical, demonstrating that there was no significant publication bias (Fig. 13).

## Discussion

To our knowledge, this is the first comprehensive meta-analysis aimed at comparing the clinical safety and efficacy between RHA and SHA on the basis of a synthesis of evidence available. The pooled results revealed that there were no differences in overall postoperative outcomes, recovery of ROM, complication rate between the two procedures. Compared to SHA, RHA could provide better pain relief but was associated with higher risk of revision. In addition, one study demonstrated that the operation time of RHA was shorter than that of SHA.

To date, TSA has been considered to be the gold standard for treating shoulder osteoarthritis due to its ability to achieve better functional improvement, clinical safety and patients’ satisfaction than HA [49, 50]. Nevertheless, HA may be preferred by some surgeons, when individual demand, expectations, age, career and pre-existing diseases are taken into account [51, 52]. Furthermore, it can also provide pain relief and avoid the glenoid loosening associated with TSA [22]. With continuous development and modification, HA has been applied for the treatment of various shoulder disorders, including the primary osteoarthritis (POA) [53], avascular necrosis (AVN) [16], rheumatoid arthritis (RA) [54], cuff-tear arthropathy (CTA) [18], juvenile idiopathic arthritis (JIA) [55] and post-traumatic degenerative joint disease (DJD) [56]. Especially, RHA has been promoted as a bone-sparing alternative to SHA for over ten years among doctors and patients. Various types of resurfacing procedures have been described in terms of short- and mid-term clinical effectiveness by mounting studies [32, 53, 57–60] and a recent systemic review [61] suggested that resurfacing replacements could provide a significant improvement in pain, motion, and standardized outcome scores.

In theory, RHA has several advantages over SHA. It preserves bone stock and restores the native anatomic structure of the glenohumeral articulation, which may render it more suitable for younger, more active population [37, 62, 63]. A cadaveric study [64] made a biomechanical comparison between RHA and SHA in terms of functional glenohumeral positions and found that resurfacing replacements could better simulate the kinematics and contact characteristics of the intact glenohumeral joint than stemmed humeral hemiarthroplasty, by preserving the anatomy of the articular surface of the humeral head. Moreover, several studies examined the safety and efficacy following RHA and SHA for different glenohumeral diseases. A Norwegian registry study [38] reported identical improvement in Oxford Shoulder Score (OSS) between 144 SHAs and 124 RHAs. Another registry study [39] compared 837 RHAs and 259 SHAs and found that RHAs obtained a significantly better WOOS scores than SHAs while there were no differences in the revision rate. Fourman *et al* [48] found that RHA was associated with less pain and clinically equivalent functional outcomes compared with SHA at mid-term follow-up. To date, whether RHA is superior to SHA remains controversial and there is no more reliable evidence to prove it.

Thus, in this study, we performed a comprehensive meta-analysis based on the currently available findings. Our study revealed identical clinical outcomes and better pain relief in the RHA group as compared to the SHA group. However, RHA carried a higher risk of revision than SHA, which was coincident with recent research results. Voorde *et al* [54] found that the revision rate was significantly higher after RHA (14%) than after SHA (2%) while two procedures yielded similar WOOS scores (61 ± 27 *vs.* 58 ± 21) in the treatment of severe RA. A single-center retrospective study including 78 patients with POAs demonstrated that survival was significantly poorer in RHA, with 4 revisions (9.8%) *verse* none in SHA (*p* < 0.05) despite similar functional scores [46]. Besides, low failure rates in the SHA group (30%) compared to the RHA group (60%) was also observed in the study by Hammond *et al* [45]. Nonetheless, we could not reach the definitive conclusion whether RHA or SHA was a better option for the treatment of shoulder diseases based on the current findings.

In addition, inevitable heterogeneity could influence consistency of the results in this meta-analysis since study design, sample size, population source, operative techniques, follow-up period and other confounding factors varied substantially among studies. Though fixed or random-effect model was used to reduce the heterogeneity and sensitivity analysis could explain the source of heterogeneity, subgroup analysis could not be conducted due to the limited number of studies or insufficient data. Several studies found some variates might affect the clinical outcomes or risk for revision between RHA and SHA. Magnus *et al* [40] found that the significant difference in risk for revision between RHA (12%) and SHA (6.7%) was related to the age of patients, and the lower age presented the higher risk of revision. Meanwhile, they also found different diagnoses could affect the functional outcomes of two procedures, and patients with POA had a better outcome than their counterparts with SOA [40]. Fourman *et al* [48] found the follow-up time exerted an effect on clinical effectiveness of RHA or SHA. After a follow-up of more than 8 years, no significant difference in patient-reported outcomes was observed between two procedures while the total ASES score was significantly better after RHA than SHA at ≧ 8 years of follow-up. Unfortunately, we could not further evaluate potential influence of certain factors through subgroup analysis. Anyway, our results might be useful for surgeons because they will try their best to preserve more bone tissue, shorten the operation time, and reduce postoperative complications.

This meta-analysis has several limitations. First, included studies involved various outcome measures and the data were insufficient in some respects. Moreover, the number of eligible studies was limited. For instance, only 2 comparative studies reported the ASES, WOOS and DASH, which might impact the accuracy of the result. In addition, we made a descriptive analysis for those important indicators reported in a single study. Second, some variables or indicators such as age, follow-up period or types of osteoarthritis could not be stratified for further subgroup analysis due to the small size of the study samples and, as a consequence, some pooled results had significant heterogeneity. Third, the follow-up duration of the included studies was inadequate, and especially they lacked long-term follow-up data in the comparison of the two procedures. Finally, publication bias and selection bias were inevitable because only English-language studies were included and quality levels varied. Given these limitations, data from the present studies must be interpreted with caution.

## Conclusion

This meta-analysis indicated that RHA was associated with more pain relief but higher revision rates when compared with SHA. However, overall patient-reported outcomes, improvement of ROM and complication rate showed no differences between the two technique. On the basis of the present evidence, it is still hard to decide if a procedure is superior to another. Therefore, treatment options should be determined carefully in accordance with individual differences. Large-sized, high-quality and well-designed RCTs with long-term follow-up are warranted to find more convincing evidence concerning the superiority of the two techniques.

## Data Availability

Not applicable.

## Abbreviations

TSA: Total shoulder arthroplasty
RSA: Reverse shoulder arthroplasty
HA: Hemiarthroplasty
HHR: Humeral head resurfacaing
RHA: Resurfacing hemiarthroplasty
SHA: Stemmed hemiarthroplasty
GHOA: Glenohumeral osteoarthritis
POA: Primary osteoarthritis
SOA: Second osteoarthritis
AVN: Avascular necrosis
RA: Rheumatoid arthritis
CTA: Cuff-tear arthropathy
JIA: Juvenile idiopathic arthritis
DJD: Degenerative joint disease
CMS: Constant-Murley score
ASES: American shoulder and elbow surgeons score
WOOS: Western Ontario Osteoarthritis of the Shoulder index
Quick-DASH: Quick-Disabilities of the Arm, Shoulder and Hand score
ROM: Range of motion
VAS: Visual analog scale
OSS: Oxford Shoulder Score
EQ-5D: EuroQol 5 dimension 3 L
ADL: Activities of daily living
SSV: Subject Shoulder Value
SANE: Single Assessment Numeric Evaluation
SST: Simple Shoulder Test
PRISMA: Preferred Reporting Items for Systematic Reviews and Meta-Analyses
MeSH: Medical Subject Headings
NOQAS: Newcastle-Ottawa Quality Assessment Scale
OR: Odds ratio
SMD: Standard mean difference
CI: Confidence intervals
SD: Standard deviations
